# Effectiveness of physical therapy in axillary web syndrome after breast cancer: a systematic review and meta-analysis

**DOI:** 10.1007/s00520-023-07666-x

**Published:** 2023-04-12

**Authors:** Jesús Baltasar González-Rubino, Maria Jesus Vinolo-Gil, Rocío Martín-Valero

**Affiliations:** 1 Department of Physiotherapy, Faculty of Health Sciences, University of Malaga, CTS-1071 Research Group, Malaga, Spain; 2 Department of Nursing and Physiotherapy, University of Cadiz, 11009 Cadiz, Spain; 3 Rehabilitation Clinical Management Unit, Interlevels-Intercenters Hospital Puerta del Mar, Hospital Puerto Real, Cadiz Bay-La Janda Health District, 11006 Cadiz, Spain; 4 Research Unit, Department Biomedical Research and Innovation Institute of Cadiz (INiBICA), Puerta del Mar University Hospital, University of Cadiz, 11009 Cadiz, Spain

**Keywords:** Breast neoplasms, Physiotherapy specialty, Lymphatic system, Range of motion, Functional status, Axillary web syndrome, Axillary lymphadenectomy

## Abstract

**Background:**

The axillary web syndrome (AWS) is a surgical breast cancer sequel that limits the functionality of the patient and delays the protocol times of application of cancer treatments. This implies a long period of discomfort and limitations for the user.

**Objective:**

To investigate the different physiotherapy treatments for the AWS and how effective they are.

**Methods:**

A systematic review based on PRISMA protocol and registered in PROSPERO (CRD42021281354) was conducted. The research was performed using PubMed, Scopus, CINAHL, PEDro, and Web of Science databases during January 2022 and March 2022. All randomized controlled trials and controlled clinical trials were included in this review.

**Results:**

A total of 188 articles were identified, with 9 studies selected for the systematic review. These studies basically propose treatments based on exercises and stretching, manual therapy, and the combination of manual therapy and exercises.

**Conclusions:**

Exercise and stretching are the most effective therapies within the field of physiotherapy for the rehabilitation of axillary web syndrome. They restore range of motion faster, reduce pain, improve quality of life, and reduce disabilities. Manual therapy, scar massage, and myofascial release could help improve outcomes but with worse results. The meta-analysis conclusion is that pain is the only outcome with a significant reduction after the application of physiotherapy treatments − 0.82 [− 1.67; 0.03]. This conclusion is drawn from the only three studies with small sample sizes.

## Introduction

Breast cancer is the most common cancer in women around the world and it is one of the leading causes of death among women in developed countries [1]. It is an important public health issue, since according to the World Health Organization, more than two million new cases are diagnosed annually worldwide, becoming almost a quarter of malignant tumors in females [2]. In the Western world, it has been shown that one in nine to twelve women will suffer from this disease in her lifetime [3].

Most cases occur in post-menopausal women and the main age at diagnosis is around 60 years [4].

After the diagnosis of breast cancer, the patient normally undergoes surgical and/or cancer treatment. Chemotherapy, radiotherapy, and hormonal therapy are some of the treatment alternatives which are currently adapted with precision to the type of tumor in order to achieve a better response and survival [5].

Post-mastectomy lymphedema is one of the best-known post-surgical and post-actinic sequelae after breast cancer, with a prevalence of around 20% of mastectomized women. Lymphedema is the swelling of the upper limb due to a lymph node dissection or axillary radiation therapy [6]. The conservative treatment of this health issue is based on decongestive physical therapy and physiotherapy. The physiotherapy technique with the best results and clinical evidence to treat lymphedema is decongestive physical therapy [7, 8]. Pneumatic multicompartmental pressotherapy helps reduce the feeling of heaviness and stiffness of edema. Pressotherapy can be incorporated into decongestive physical therapy in the treatment of some lymphedema to help reduce edema [9].

In addition to post-mastectomy lymphedema, the patient undergoing surgery for breast cancer may present axillary web syndrome (AWS) or superficial lymphatic thrombosis. AWS is a surgical sequel after breast cancer independent of lymphedema. They have both different clinical manifestations and evolution times. Lymphedema is a chronic health problem while AWS has a spontaneous evolution time of 3 months. They may appear at the same time or independently*.* As described by Yeung et al. in their systematic review [10], AWS can appear in the first 8 weeks after the operation and usually resolves spontaneously within 3 months of its appearance [11].

The lymphatic thrombus is clinically manifested as a cord that frequently occurs in the armpit, although it can also appear along the upper limb, elbow crease even reaching the first finger [11–13]. Regarding the diagnosis through imaging tests, nuclear magnetic resonance does not manage to clearly identify the axillary network syndrome. Ultrasound, on the other hand, is the most reliable method as it allows movement to the patient’s arm while the diagnostic test is being carried out [12, 14].

The axillary web syndrome produces pain when abducting and flexing the shoulder with the respective loss of functionality and limitation of mobility of the affected upper limb [15].

According to the American Cancer Society, radiation therapy is applied 3–8 weeks after the operation if chemotherapy is not required. If chemotherapy is used, it is applied 3–4 weeks after completion. It is usually applied 5 days a week from Monday to Friday.

The limitation of mobility often leads to a delay in the application of this useful tool in the oncological therapeutic arsenal to prevent recurrences [16–18]. Hence, the need and importance of this review. The main objective is to investigate the different physiotherapy treatments for the AWS and how effective they are.

The frequency of the AWS is not clear from the current publications. It depends on the type of surgical intervention, age, BMI [19], the appearance of the post-operative seroma, and even breast reconstruction [20]. Thus, being 30% the frequency of the operated patients [11, 21].

After reviewing the relevant literature, it is aimed to systematically retrieve, appraise, and synthesize findings to management the AWS as well as provide a summary of current evidence to inform subsequent research in this field.

## Material and methods

This study was carried out according to the Preferred Reporting Items for Systematic Reviews and Meta-Analyses Statement (PRISMA). The protocol was registered in PROSPERO (CRD42021281354). As this was a literature review, it did not involve the recruitment of subjects and it analyzed data from already published original articles; however, this study has the approval of the Andalucía Ethics Committee with PEIBA code 1909-N1-21 reg. number 171.21. Searches began on the first of January 2022 and ended with the last search query in March 2022. The details of the search strategy used are reported in Table 1. We explored grey literature sources but its results were not considered. We adopted a broad search methodology to ensure the maximum inclusion of studies reporting both outcomes. After the preliminary identification, the articles were exported and managed in Rayyan QCRI (https://www.rayyan.ai/).

**Table 1:** Detailed search strategy in PubMed, Scopus, CINAHL, PEDro, and Web of Science

Database	Search strategy
PubMed	(axilla* web syndrom* OR axilla web OR axilla* cord* OR axilla string OR axilla* band*) AND (breast cancer OR breast neoplasm OR breast carcinoma OR breast tumour) AND (physiotherap* OR rehabilitation OR physical therapy)
Scopus	TITLE-ABS-KEY [((axillary AND web AND syndrome) OR (axilla AND string) OR (axilla AND band) OR (axilla AND cord)) AND (physiotherapy)]
CINAHL	(axillary web syndrome AND physiotherapy OR axillary web syndrome AND stretching OR physiotherapy AND lymphatic AND thrombus)
PEDro	((axillary web syndrome AND string AND band) OR (axillary web syndrome OR string OR band))
Web of Science	(axilla* web syndrom* OR axilla web OR axilla* cord* OR axilla string OR axilla* band*) AND (breast cancer OR breast neoplasm OR breast carcinoma OR breast tumour) AND (physiotherap* OR rehabilitation or physical therapy)

Reference lists of relevant studies selected for the review were manually searched for further studies that may meet the study selection criteria. If it was not clear, the full text was reviewed.

The restrictions were applied for language (English, Spanish, German) and year of publication (March 2012 until March 2022). Studies published in the last 10 years were selected. There were no restrictions applied for type of publication. Two independent reviewers (JBGR and MJVG) conducted the search using the same methodology, and the differences were resolved by consensus moderated by a third reviewer (RMV). We used Rayyan software to organize studies, assess studies for eligibility, and remove duplicates.

### Eligibility criteria

Eligible characteristics were based on different items of the PICO scheme (Population, Intervention, Comparison, Outcomes) [22]. Overall, selection criteria were kept general at the outset to provide an overview of the topic and to ensure finding all relevant sources. Further specific narrowing was carried out in a later phase.

Studies that recruited women above 18 years old, who had suffered from breast cancer, undergone surgical intervention, with radical or conservative surgery and with axillary lymph node dissection or with the sentinel node technique. All subjects must present the lymphatic thrombus. In order to diagnose lymphatic thrombus, it must be palpable, visible, and must limit the mobility and functionality of the arm. Other characteristics such as age, socioeconomic, and ethnic characteristics of the women were not further considered or narrowed down.

Interventions that were eligible for inclusion had to include physical therapy or rehabilitation treatments. Anthropometric characteristics of the enrolled participants were considered. These could be exercises, stretching, different types of manipulative therapy, or massage. The therapy could be applied by an experienced therapist or by the previously trained patient herself. Type of physical therapy, duration, guidance, and the number of sessions must be defined to provide a comprehensive overview in the AWS field.

All randomized controlled trials and controlled clinical trials were included in this review.

All duplicate titles were removed. All authors independently screened titles and abstract to remove manuscripts that did not meet the inclusion criteria. Studies were included if they were written in English, Spanish, or German. Eligible studies included trials on AWS.

The studies associated with Mondor’s disease, case control studies, case studies, systematic review, and low methodological quality studies were excluded.

Full text of the remaining studies was then retrieved and reviewed independently by all authors to confirm eligibility for inclusion. Justifications for excluding studies were noted and discrepancies between authors were discussed. Authors reached consensus.

To measure the change of the clinical symptoms of AWS after rehabilitation and physical therapy (quality of life, arm disability, pain, ROM, strength, and limb volume) are the outcome measures of the intervention which are considered.

Quality assessment for the selected studies was conducted by using the Physiotherapy Evidence Database (PEDro) Scale [10, 23]. This assessment tool is an elaborated instrument for assessing the quality of a physical therapy study but also it can be used in different health-related topics. It includes several items which help classifying studies according to their methodological quality. The PEDro scale was developed to help PEDro users to rapidly identify trials that are likely to be internally valid and have sufficient statistical information to guide clinical decision-making. Each trial study is given a total PEDro score, which ranges from 0 to 10. Total PEDro scores of 0–3 are considered “poor methodological quality,” 4–5 “fair methodological quality,” 6–8 “good methodological quality,” and 9–10 “excellent methodological quality.” It considers selection bias, design, blinding, data collection methods, and withdrawal and dropouts.

### Assessment of quality

This study adopted the Cochrane risk bias assessment approach for evaluating the methodological quality of all enrolled articles. It evaluated the generation of random sequences, concealment of allocation, participant/personnel blinding, outcome measure blinding, selective reporting, insufficient outcome data, and additional biases involved in those articles. Each item was rated as “yes,” “no,” or “unclear.”

The risk of bias was calculated for each study selected using the Cochrane Collaboration Tool [24]. The following types of bias were assessed: selection bias, performance bias, detection bias, attrition bias, reporting bias, and other bias. Two reviewers (J.B.G.R and M.J.V.G) assessed the methodological quality and the risk of bias of the studies. In case of doubt, authors resolved disagreements by consensus and consulted a third author (R.M.V.) when necessary.

### Data extraction and data analysis

Data extraction was performed in a standardized manner, with each author examining an equal number of studies individually and extracting all relevant information from the respective studies into spreadsheet format. Based on the results of data extraction, the authors discussed ambiguities and jointly decided which studies should lastly be included for analysis in the review.

### Statistical analysis

Related outcome variables were imported into Review Manager (Version 5.4.1, The Cochrane Collaboration, 2020) for meta-analysis. Continuous outcome variables were examined for all the enrolled articles. The mean difference (MD) was chosen as the effect scale index for identical test methods and units, while the standardized mean difference (SMD) was selected otherwise. Moreover, this study adopted the *I*_2_ statistic for analyzing heterogeneity among diverse articles, where *I*_2_ < 50% represented the absence of heterogeneity, in which case a fixed effects model was applied. Finally, a funnel plot was drawn to check the possible bias among articles, and a forest plot was adopted to determine MD and SMD.

## Results

### Search results

**Fig. 1:**
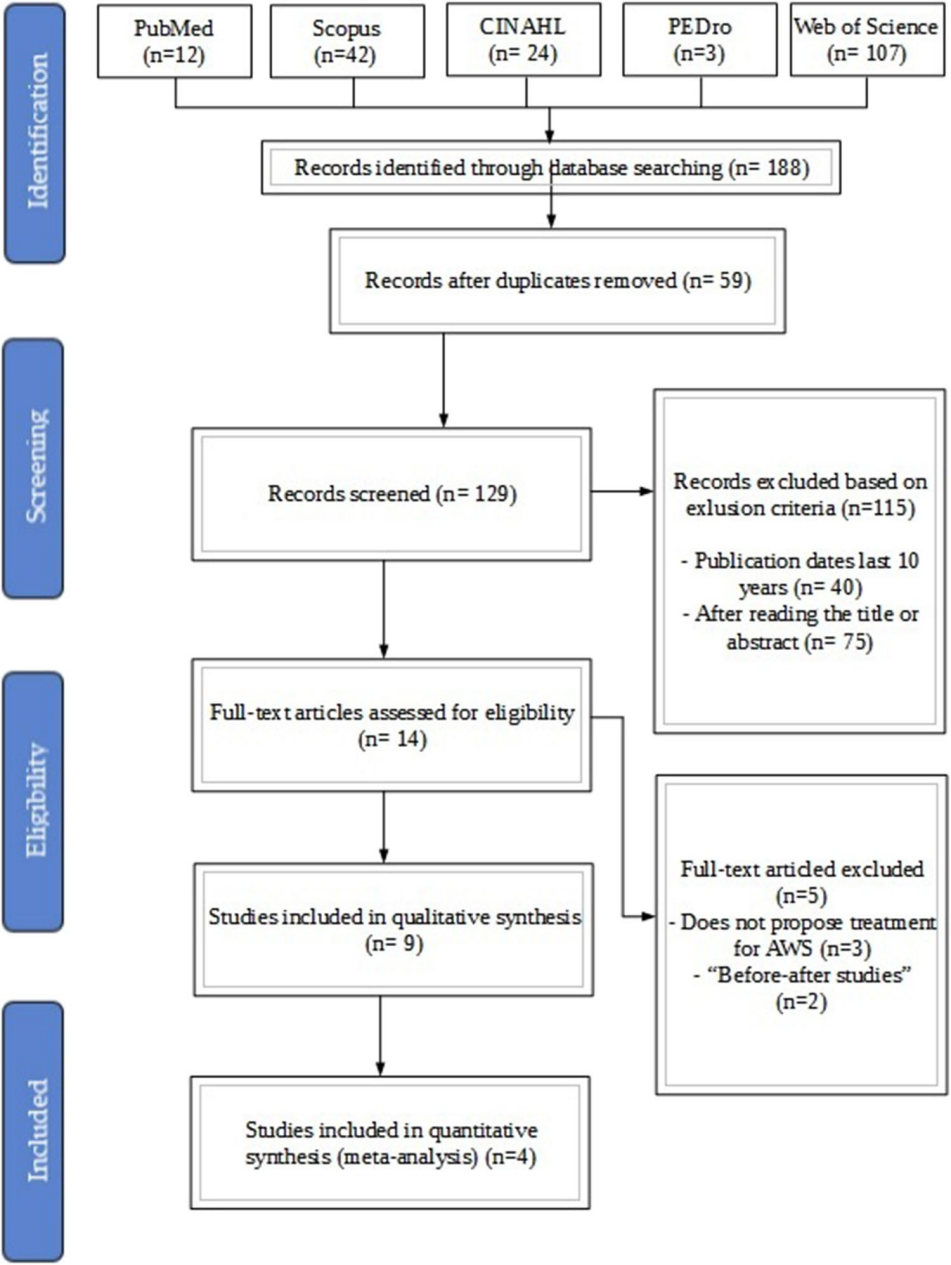
PRISMA flow diagram of the study selection process

The first search obtained 188 studies which were filtered by title. After the duplicated studies were removed, 129 studies were retrieved. After screening the title and abstract, 115 studies were rejected. Details can be observed in Fig. 1.

### Study design and intervention characteristics

Nine clinical trials have been reviewed, and it can be seen that a total of 661 subjects were evaluated in the studies selected. The article that used the largest sample size was Klein et al., 2021 [25], with 157 subjects, while the one that used the smallest sample size was Nimirti et al., 2019 [26], with 10 patients. With regard to the characteristics of the participants, all of them were women older than 18 years old. They were operated of breast cancer with conservative or radical surgery, and with axillary lymph node dissection or with sentinel node biopsy.

Three of the articles had Spanish authorship, one Korean, one Polish, one Indian, one Canadian, one Egyptian, and one from Israel.

According to the intervention carried out, seven studies used manual lymphatic drainage and exercises as the main treatment for AWS [26–28], two studies used myofascial release as the main treatment [26, 29], two studies used exercises as the main treatment [30, 31], and one study used exercises and scar treatment as the main treatment [25].

The study with the shortest duration was 3 weeks [26, 27] and the longest was 54 weeks [32]. In Table 4, the different study variables analyzed can be observed. The most evaluated variables were ROM [25, 27–31], strength [25, 28, 31], quality of life [25–28, 32], pain [25, 27–30, 33], and the shoulder disabilities [25, 27–30].

In order to estimate and compare the effectiveness of the interventions, the improval was calculated using validated scales such as EORTC QLQ-BR-23/BR-30 for the quality of life [25, 26, 28, 32]; DASH [28–30], OSS [27], or SPADI [25] for the arm disability; and VAS [27, 29, 33] or NPRS [28, 30] for the pain. Goniometry [25, 29, 31], the application Dr Goniometer [30], or inclinometer [27, 28] were used to measure ROM; the handgrip dynamometer JAMAR [31] or POWER TRACK II COMMANDER [28] for strength; and circometry combined with the simplified water displacement technique [28, 32] was used for the volume of the upper limbs [34].

The patients who belonged to the control groups received basic medical recommendations with provision of general information and encouragement on maintaining a healthy active lifestyle with a brochure [25] or by a professional [26, 27, 31]. Two studies did not have a control group, but in both groups, there were different interventions [28, 33]. The patients in the control group of another study received nursing recommendations [30]; in another, the control group received a standardized physical exercise program [32]; and in another, they received stretching with moist heat [29].

Regarding the duration of the treatment sessions in the intervention group, six studies specified the time of the physiotherapy treatment sessions or the number of repetitions of each exercise in a very specific way (intervention group) [25–28, 31, 32]. Three studies did not detail the duration of the intervention group sessions [29, 30, 33].

The best results obtained in the analyzed studies were for the variables pain [25, 27–31, 33], ROM [25, 27, 29, 31], functionality [27–30], and quality of life [25–28] (especially in functional aspects of health-related quality of life) [27]. There was no strong statistical evidence found for other dimensions of quality of life [26]. One study did not find a significant difference between groups in health-related quality of life [32].

One study obtained good results in reducing the thickness of the cord [33].

Significant and clinically relevant differences between groups were found at the 3-month follow-up. No significant differences were found at the 6-month follow-up [27, 31].

Table 2 summarizes the studies included in this systematic review investigating the efficacy of physiotherapy treatments for the AWS after breast cancer.

**Table 2:** PICO table

Study	*N*/age	Intervention	Duration/sessions	Outcome measure/measuring instrument	Results
Gamal et al., 2018 [33]	60/40–50 years	*IG* -Myofascial release and kinesio tape *IG* -Myofascial release *IG* -Kinesio tape	4 weeks/2 per week	-Pain (VAS) -Thickness of the cord (ultrasound) -Cord disorganization (ultrasound)	-Myofascial release + kinesio tape group decreases VAS value (*p* = 0.00001) pre-intervention 6.30 ± 1.03 post-intervention 1.25 ± 0.63 -Decrease in thickness of the cord (*p* = 0.0001) pre-intervention 0.31 ± 0.17 post-intervention 0.24 ± 0.09 -Improvement in the cord disorganization (*p* = 0.003) marginal homogeneity test value = 24.5
Ibrahim et al., 2018 [31]	59/18–45 years	*IG* -Exercises for mobility, strength, and endurance *CG* -Standard care -Provision of general information -Healthy and active lifestyle	12 weeks	-Clinical characteristics -Shoulder ROM (goniometer) -Handgrip strength (Jamar hand dynamometer)	ROM limitations for abduction at baseline *p* < 0.0196 (from 10.7° to 18.5°), external rotation *p* < 0.0475 (from 2.6° to 6.0°), horizontal rotation *p* < 0.0327 (from 0.8° to 8.5°) 18 months after radiation, flexion *p* < 0.0216, (from 5.4° to 9.1°)
Klein et al., 2021 [30]	157/18–85 years	*IG* -Therapeutic exercises -Instructions for home exercise *CG* -Information about pain, wound care, and instructions for home care	6 months	-Pain (NPRS) -Disabilities (QuickDASH) -ROM (DrGoniometer application)	CG pain levels: NPRS 2.1 ± 1.4 (*p* = 0.011) Early PT reduces pain: NPRS 0.5 ± 0.8 (*p* = 0.019) Positive effect on functional disabilities: -Small surgeries *p* = 0.004 CG 10.0 ± 12.0 IG 5.0 ± 7.0 -Extensive surgeries *p* = 0.032 CG 5.0 ± 7.0 IG 1.1 ± 2.0
Muñoz et al., 2021 [25]	40/18–90 years	*IG* -Functional recovery exercises -Scar treatment *CG* -Usual care (recommendations in written form)	1 month/4–6 sessions in total	-ROM (goniometer) -Strength (Dinamometer JAMAR) -Pain and disability (SPADI) -State of scar (POSAS) -Tissue adhesions (MAP-BC evaluation tool) -Quality of life (EORTC QLQ-BR-23) -AWS (Observation/palpation)	-Significant improvements in experimental group: Global shoulder ROM (*p* = 0.003) Pre-intervention 94.87 (26.92) Post-intervention 100.00 (0.00) Global SPADI (*p* = 0.001) Pre-intervention 32.30 (26.84) Post-intervention 14.55 (14.95) State of scar (*p* = 0.000) Pre-intervention 25.90 (9.57) Post-intervention 9.00 (5.60) Tissue adhesions (*p* = 0.000) Pre-intervention 28.15 (9.66) Post-intervention 5.70 (5.33) Quality of life (*p* = 0.013) Pre-intervention 47.71 (11.29) Post-intervention 53.72 (12.55)
Nimirti et al., 2019 [29]	10/	*IG* -Myofascial release -Moist heat *CG* -Conventional treatment (stretching and moist heat)	4 weeks/3 per week	-Pain (VAS) -Disabilities (DASH) -ROM (goniometry)	-Significant improvement post-treatment in experimental group: shoulder flexion (*p* = 0.012) CG 138 ± 19.23 IG 168 ± 8.36 Extension (*p* = 0.049) CG 46 ± 4.18 IG 54 ± 6.51 Abduction (*p* = 0.041) CG 139 ± 23.02 IG 166 ± 9.61 Internal rotation (*p* = 0.006) CG 49 ± 5.47 IG 71 ± 12.45 External rotation (*p* = 0.012) CG 61 ± 7.41 IG 76 ± 7.41 DASH (*p* = 0.045) CG 30.16 ± 6.13 IG 21.72 ± 5.10 VAS (*p* = 0.047) CG 3.40 ± 0.547 IG 2.0 ± 1.22
Ochalek et al., 2017 [32]	45/	*IG* -Circular-knit compression class I sleeve garment -Physical activity -Standard educational leaflets *CG* -Physical activity -Standard educational leaflets	54 weeks/7 per week	-Quality of life (EORTC QLQ-C30 and QLQ-BR23) -Limb volume (Circometry, Frustum formula)	15–21 mmHg sleeves + physical activity prevents lymphedema (*p* < 0.001) Edema volume CG 114.5 (14.9–166.5) IG − 67.6 (− 144.1 to − 24.2)
Torres et al., 2022 [27]	96/	*IG* -Physical therapy -MLD -Active arm exercises *CG* -Standard arm exercises (proprioceptive neuromuscular exercises) -Usual-care practice	3 weeks/3 per week	-Pain (VAS) -ROM (digital inclinometer) -Shoulder disability (OSS) -Quality of life (FACT-B)	Pain intensity significantly decreased (*p* < 0.001) Mean difference 95%CI: − 14.22 (− 15.53 to − 12.90) Shoulder ROM at 3-month follow-up (*p* < 0.001) Mean difference 95%CI: 38.36 (31.91 to 44.81) Shoulder disability significantly improved (*p* < 0.001) Mean difference 95%CI: 6.09 (5.51 to 6.68)
Cho et al., 2016 [28]	41/	*IG* -Physical therapy -MLD *IG* -Physical therapy	4 weeks/3–5 per week	-ROM (digital inclinometer) -Limb volume (circometry + formula) -Strength (dynamometer Power Track II Commander) -Quality of life (EORTC QLQ-C30 and QLQ-BR23) -Arm disability (DASH) -Pain (NPRS) -AWS (visible/palpable)	DASH score (*p* = 0.000) Mean difference 95%CI 15.7 ± 10.6 NPRS score (*p* = 0.000) Mean difference 95%CI 1.5 ± 1.0 EORTC QLQ-C30 (*p* = 0.000) Mean difference 95%CI 23.3 ± 15.7 EORTC QLQ-BR23 (*p* = 0.000) Mean difference 95%CI 66.2 ± 16.6 ROM flexion and abduction (*p* = 0.000) Mean difference 95%CI 180 ± 0.0
Yuste et al., 2014 [26]	153/ > 18 years	*IG* -MLD -Kinesiotherapy -Scar massage -Proprioceptive Neuromuscular facilitation exercise -Therapeutic education program *CG* -Therapeutic education program	3 weeks/3 per week	-Quality of life (EORTC QLQ-BR23)	(12 months post-surgery) EORTC QLQ-BR23 (*p* > 0.05) no strong statistical evidence was found except for: Physical function (*p* = 0.00295) 8.6 (3.5 to 13.8) Social function (*p* = 0.00298) 4.2 (− 4.2 to 12.6)

*N*, number or participants; *MLD*, manual lymphatic drainage; *PT*, physical therapy; *CG*, control group; *IG*, intervention group; *mm Hg*, millimeters of mercury

Instruments: *VAS*, Visual Analogue Scale; *ROM*, Range of Movement; *NPRS*, Numeric Pain Rating Scale; *DASH*, Disabilities of Arm, Shoulder and Hand; *SPADI*, Shoulder Pain and Disability Index; *POSAS*, The Patient and Observer Assessment Scale; *MAP-BC*, Myofascial Adhesions in Patients after Breast Cancer; *EORTCQLQ-BR-23*, European Organization for Research and Treatment of Cancer Quality of Life Questionnaire; *OSS*, Oxford Shoulder Score; *FACT-B*, Functional Assessment of Can; *CI*, confidence interval

PEDro critical appraisal checklist was applied to the 9 remaining studies (Table 3). Five studies included criteria validity, which were inconsistently rated very good [26, 27, 30, 32, 33] and 4 were rated doubtful methodological quality [25, 28, 29, 31] (Table 4).

**Table 3:** Study variables

Study	ROM	Handgrip strength	Quality of life	Pain	Arm disabilities	Arm volume	AWS presence	State of the scar	Tissue adhesions	Disorganization and Thickness of the cord
Torres et al., 2022 [27]	x	-	x	x	x	-	-	-	-	-
Muñoz et al., 2021 [24]	x	x	x	x	x	-	x	x	x	-
Klein et al., 2021 [30]	x	-	-	x	x	-	-	-	-	-
Nirmiti et al., 2019 [30]	x	-	-	x	x	-	-	-	-	-
Gamal et al., 2018 [33]	-	-	-	x	-	-	-	-	-	x
Ibrahim et al., 2018 [31]	x	x	-	-	-	-	-	-	-	-
Ochalek et al., 2017 [27]	-	-	x	-	-	x	-	-	-	-
Cho et al., 2016 [28]	x	x	x	x	x	x	x	-	-	-
Yuste, 2015 [26]	-	-	x	-	-	-	-	-	-	-

The symbol “x” indicates those items that have been scored. The symbol “-” indicates those items that have not been scored

**Table 4:** PEDro scale

Study	Total score	1	2	3	4	5	6	7	8	9	10	11
Torres et al., 2022 [27]	6/10	-	x	x	-	-	-	-	x	x	x	x
Muñoz et al., 2021 [24]	4/10	-	-	-	x	-	-	-	-	x	x	x
Klein et al., 2021 [30]	7/10	-	x	x	x	-	-	-	x	x	x	x
Nirmiti et al., 2019 [30]	5/10	-	x	-	-	-	-	-	x	x	x	x
Gamal Abd et al., 2018 [33]	6/10	-	x	-	x	-	-	-	x	x	x	x
Ibrahim et al., 2018 [31]	5/10	-	x	-	x	-	-	-	-	x	x	x
Ochalek et al., 2017 [27]	7/10	-	x	-	x	-	-	x	x	x	x	x
Cho et al., 2016 [28]	5/10	-	x	-	-	-	-	x		x	x	x
Yuste, 2015 [26]	6/ 10	-	x	x	-	-	-	x		x	x	x

The symbol “x” indicates those items that have been scored; the symbol “-” indicates those items that were not counted for the final score

### Quality assessment

Two independent reviewers (JBGR and MJVG) conducted the search using the same methodology. They rated with PEDro scale each full text individually at first. Results of the individual ratings were then discussed by all authors together and in case of any ambiguities, the differences were resolved by consensus moderated by a third reviewer (RMV). Full-text analysis, data extraction, and quality assessment were completed.

### Risk of bias of included studies

**Fig. 2:**
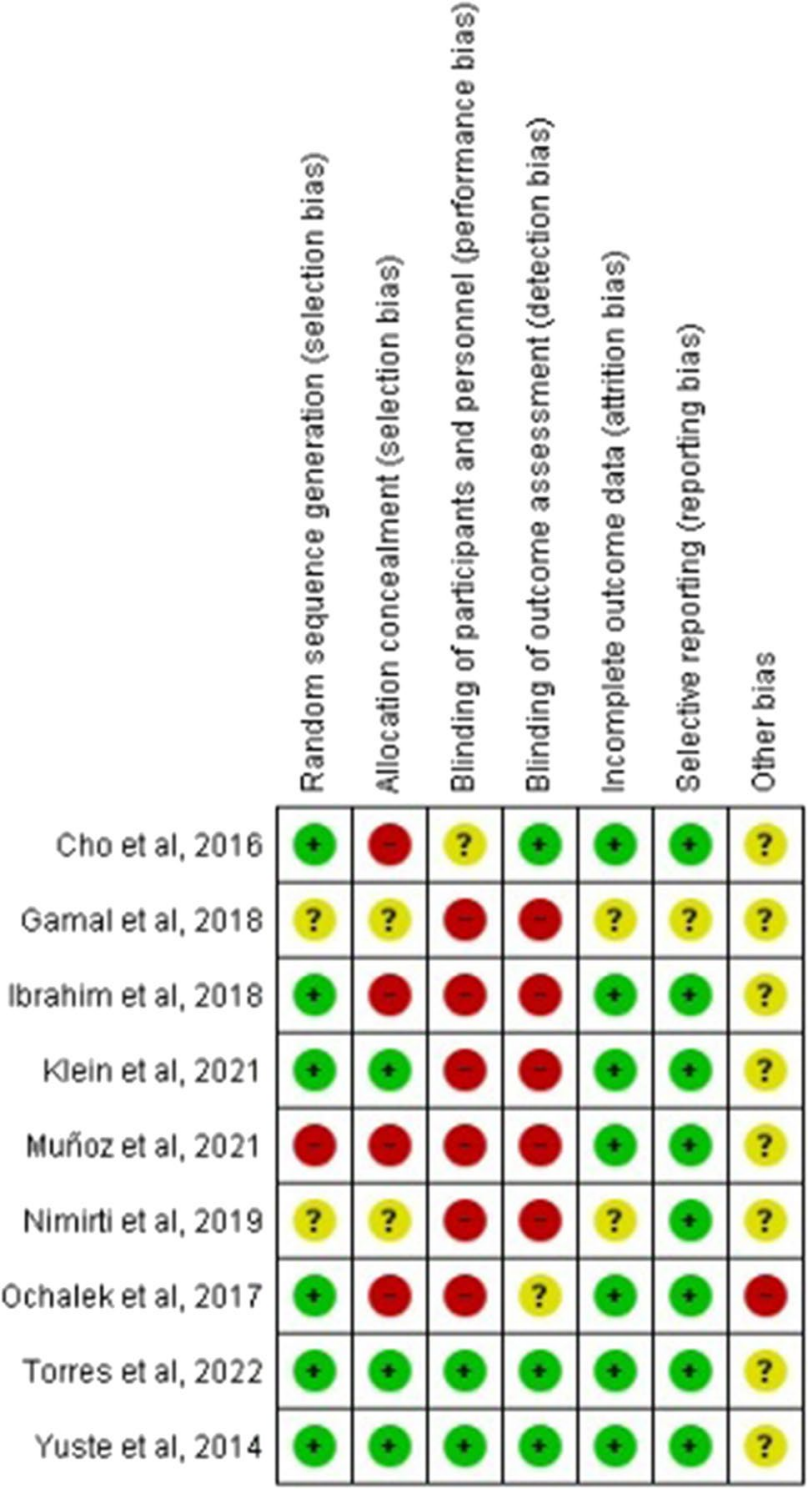
Risk of bias summary [25–33]

The Cochrane Risk of Bias Assessment Tool [35] was used to assess the risk of bias of the articles included in this review. The results of the risk of bias can be observed in Fig. 2.

**Fig. 3:**
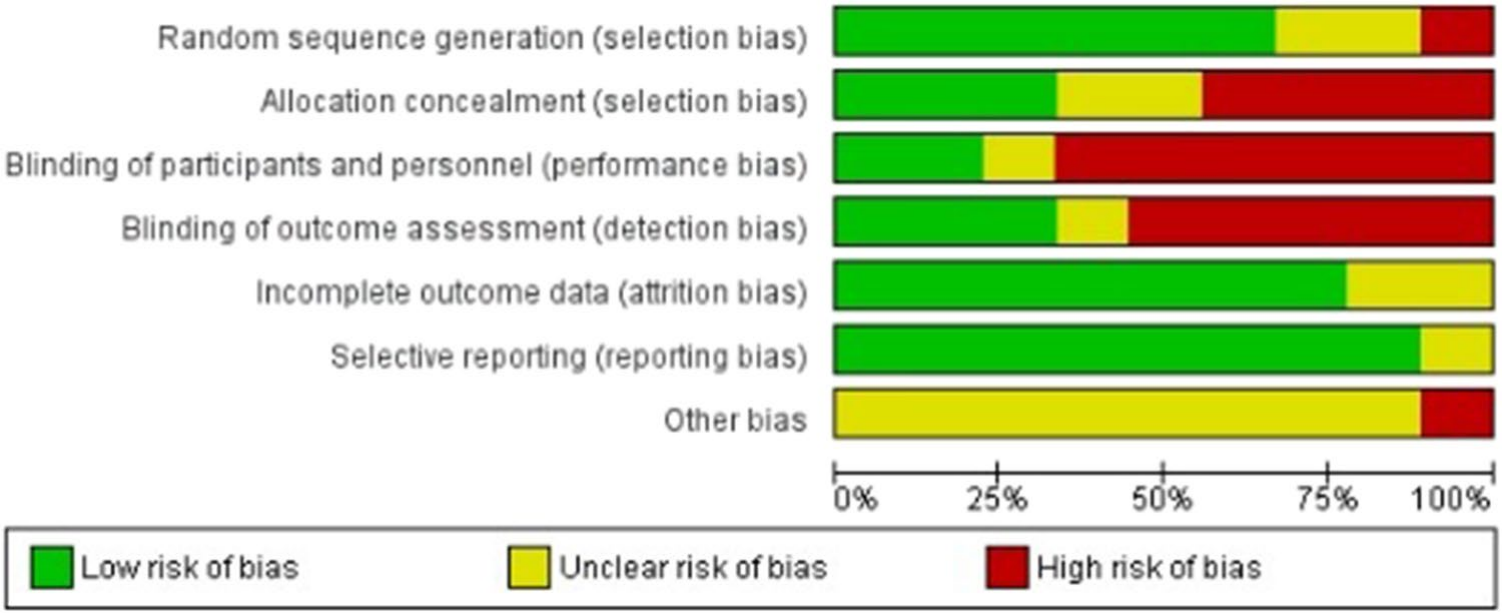
Risk of bias graph

With respect to attrition bias and reporting bias, it should be noted that the risk of bias is high in relation to “blinding of participants and personnel,” as it is difficult to perform blinding in a clinical trial where there is a physiotherapy intervention. There is also a high risk of bias in relation to “blinding of outcome assessment”: the knowledge of the allocated interventions was not adequately prevented during the study, because the researcher who carried out the intervention was the same one who extracted the data (Fig. 3).

### Meta-analysis

#### Outcome pain

Three studies measured pain or some pain dimension for up to 6 months [25, 29, 30].

Studies that used VAS scale or SPADI scale for pain outcome were considered for our meta-analysis.

One study [27] was discarded because it did not show the mean or the standard deviation of this variable for the control group in their results. Information from the corresponding author was requested regarding this issue but we received no reply from them.

There was a significant difference in these studies regarding the interventions and the variable pain (overall random effects, 95%CI − 0.82 [− 1.67; 0.03]).

**Fig. 4:**
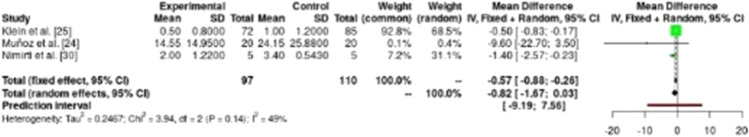
Forest diagrams of the meta-analyses of those studies evaluating the effect of the treatment compared to the control group in relation to the pain at the end of the treatment (meta-analysis involves the clinical trials’ short-term results)

The heterogeneity index is moderate (*I*_2_ = 49%). As studies are not very similar, results should be interpreted with some caution (Fig. 4).

#### Outcome ROM-Abduction

Four studies measured abduction of the shoulder for up to 6 months [25, 28–30].

Studies that used goniometry for ROM outcome were considered for our meta-analysis.

Two studies [27, 31] were discarded because they did not show the mean or the standard deviation of this variable for the control group in their results. Information from the corresponding author was requested regarding this issue but we received no reply from them.

There was not a significative difference in these studies regarding the interventions and the variable ROM-Abduction (overall random effects, 95%CI − 1.08 [0.92; 1.27])).

**Fig. 5:**
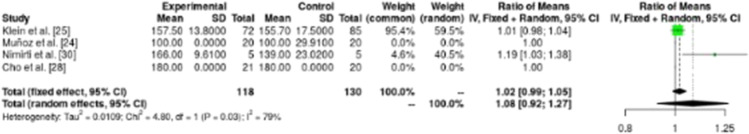
Forest diagrams of the meta-analyses of those studies evaluating the effect of the treatment compared to the control group in relation to the abduction of the shoulder at the end of the treatment (meta-analysis involves the clinical trials’ short-term results)

The heterogeneity index is very high (*I*_2_ = 79%). The studies are very disparate, which makes it difficult to express results in quantitative data (Fig. 5).

#### Outcome ROM-Flexion

Four studies measured flexion of the shoulder for up to 6 months [25, 28–30].

Studies that used goniometry for ROM outcome were considered for our meta-analysis.

Two studies [27, 31] were discarded because they did not show the mean or standard deviation of this variable for the control group in their results.

There was a significative difference in these studies regarding the interventions and the variable pain (Overall random effects, 95%CI 15.49 [− 9.71; 40.68]).

**Fig. 6:**
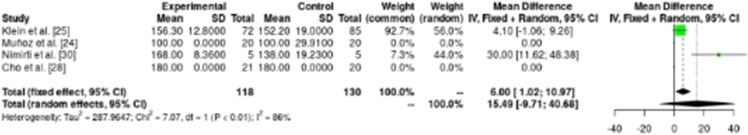
Forest diagrams of the meta-analyses of those studies evaluating the effect of the treatment compared to the control group in relation to the flexion of the shoulder at the end of the treatment (meta-analysis involves the clinical trials’ short-term results)

The heterogeneity index is very high (*I*_2_ = 86%). The studies are very disparate, which makes it difficult to express results in quantitative data (Fig. 6).

## Discussion

The objective of this systematic review was to investigate the different physiotherapy treatments for the AWS and how effective they were. This review provides a compilation of current evidence regarding the physiotherapy treatments for de axillary web syndrome AWS. After performing the analysis of the articles selected, some considerations about the studies included in this paper need to be added.

Control and intervention groups in clinical trials should be homogeneous at the beginning of the intervention. As the sample sizes of the trials analyzed are small, randomness may not have performed its function, therefore not having similar groups at the beginning. Only few studies find that the control and intervention groups have similar BMI, age, types of breast and axillary surgery, or receive similar oncology and/or radiotherapy treatments [32].

In order to make a correct diagnosis and better understanding of the etiological mechanisms of AWS, more research is needed on clinical behavior, etiopathogenic mechanisms, evolution time, and possible recurrences. Understanding why and how it occurs from the histological point of view can facilitate devising new physiotherapy treatment strategies. For these reasons, studies where biopsies are made to analyze the AWS histologically [27] should be carried out. Likewise, these studies should include a broader follow-up of the patients to observe beyond the initial 6 months from the AWS appearance [25, 27, 29] and thus, have a broader perspective. Even in the event of AWS has disappearance, it would be interesting to check if the benefits obtained with the different physiotherapy treatments are maintained or if the problem became recurrent [31, 32]. Several authors mention the benefits of acting early in the immediate post-operative period [25] and the importance of reducing initial acute pain to prevent long-term chronic pain [27].

When performing an intervention on an arm susceptible to developing lymphedema, all studies should include the volume measurement of both arms in their variables, and only 2 trials consider limb volumetry [28, 32].

Most studies use functional scales or quality of life assessment scales as outcome variables [25–30]. These scales are not considered specific to the axillary thrombus; these do not specify about its thickness, its length, the specific area where it is located, the degree of adherence, and the number of cords that may be present. For this reason, it would be interesting to provide a specific scale for AWS that would describe the characteristics of the cord and assess the changes directly in the cord produced by the physiotherapy treatment [10]. Only the study by Muñoz et al. [25] uses a scale to assess myofascial adhesions in patients who have suffered breast cancer (MAP-BC: Myofascial Adhesions in Patients after Breast Cancer). Another study used ultrasound (Samsung medisone R5 ultrasound with a high frequency probe 7–12 MHz) to compare cord thickness before and after treatment [33]. The myofascial adhesions and cord thickness are specific characteristics of the cord and its improvement can be observed directly, without having to do it through the limitations it produces.

Several studies observe improvement with the intervention of physiotherapy in the short term, but this improvement is not maintained in the long term [27, 31]. When the physiotherapy intervention ends, the benefits obtained last for a short period of time. It would be interesting for future research to try to perpetuate this improvement with exercises or longer patient follow-up.

The patients’ follow-up must be done periodically, as often as necessary and continuously to verify that they do the exercises or the recommendations correctly. Few studies adhere to the above [28, 30, 31], and if they do, they do not describe precisely details about the degree of adherence to treatment of each group during the study [26, 27].

Some studies describe a “pop” before manipulative therapy, describing it as a possible dissolution of the thrombus or release of adhesion. The explanations in this regard are conjectures and suspicions of the researcher, without having a solid pathological basis which should be developed in the future [10, 33].

Due to the diversity of scales used to measure outcome variables, only few studies could be considered for meta-analysis. In addition, due to the high rate of heterogeneity, those results must be interpreted with caution due to possible bias.

There are two systematic reviews similar to this one [10, 36], but they lack meta-analysis. The lack of clinical trials and the small sample sizes of the existing trials make their data doubtful.

Some studies [27–30] were discarded because they did not show the mean or the standard deviation of variables for the control group in their results. Information from the corresponding author was requested regarding this issue but we received no reply from them.

The study by Muñoz et al. [25] considers the ROM variable globally, without differentiating between flexion and abduction. Therefore, the ROM value is considered for both abduction and flexion.

Meta-analysis of other outcomes could not be composed due to the great diversity of scales used in clinical trials.

Pain is the only outcome with a significant reduction after the application of physiotherapy treatments. This conclusion is drawn from only three studies with small sample sizes. Hence, its moderate index of heterogeneity.

In this review, some limitations should be mentioned. Some of the studies included in the review did not have a control group in which no intervention was performed, but conventional care was practiced, in order to have the approval of the ethics committee [26, 28, 33]. It would be highly recommended to develop randomized clinical trials with a control group without intervention to be able to compare with the intervention group.

The studies analyzed have a high risk of bias due to the difficulty of blinding the intervention, the evaluator, the patient, and the therapist, as there are not many physiotherapists specialized in lymphology in the same health center.

The sample size of the studies is usually quite small [25, 28, 29, 32] due to insufficient number of patients or excessive patient dropouts [32]. For this reason, the number of patients participating in clinical trials should be increased as well as including multicenter studies [28], avoiding biased samples.

Finally, it should be taken into account for future clinical trials to specify in a very clear graphic way the intervention, these may include pictures. Exercises and stretching should be explained in a comprehensible way to help future therapists in the performance of the intervention and to obtain similar results with larger patient samples [26].

## Review limitations

This review has limitations that must be considered. One of the main limitations to the interpretation of findings from this review is the risk of bias in the included studies, due to the lack of randomized clinical trials with large sample sizes and good design. The authors of this review considered that most included studies have methods susceptible to bias.

## Future research

It is necessary to carry out blinded randomized clinical trials, with a good design and a large sample size, to avoid risk of bias.

It is also important to objectively define and diagnose AWS with the help of a scale in order to classify its characteristics.

## Conclusions

Exercise and stretching are the most effective therapies within the field of physiotherapy for the rehabilitation of axillary web syndrome. They restore ROM faster, reduce pain, improve quality of life, and reduce disabilities. Manual therapy, scar massage, and myofascial release could help improve outcomes but with worse results.

There is a clear need for further rigorous research in this field. Well-designed, randomized studies with larger sample sizes are needed to provide more conclusive data to inform best practice for the management of axillary web syndrome in rehabilitation treatments.

## Data Availability

Data available on request due to privacy/ethical restrictions.
