# Correction to: Effectiveness of physical therapy in axillary web syndrome after breast cancer: a systematic review and meta‑analysis

**DOI:** 10.1007/s00520-024-08993-3

**Published:** 2024-11-12

**Authors:** Jesús Baltasar González‑Rubino, Maria Jesus Vinolo‑Gil, Rocío Martín‑Valero

**Affiliations:** 1 Department of Physiotherapy, Faculty of Health Sciences, University of Malaga, CTS-1071 Research Group, Malaga, Spain; 2 Department of Nursing and Physiotherapy, University of Cadiz, 11009 Cadiz, Spain; 3 Rehabilitation Clinical Management Unit, Interlevels‑Intercenters Hospital Puerta del Mar, Hospital Puerto Real, Cadiz Bay-La Janda Health District, 11006 Cadiz, Spain; 4 Research Unit, Department Biomedical Research and Innovation Institute of Cadiz (INiBICA), Puerta del Mar University Hospital, University of Cadiz, 11009 Cadiz, Spain

**Correction to: Supportive Care in Cancer (2023) 31:257**

10.1007/s00520-023-07666-x

- This is the missing bibliographic reference in the article by Torres et al.:
  *Torres-Lacomba M, Prieto-Gómez V, Arranz-Martín B, Ferrandez JC, Yuste-Sánchez MJ, Navarro-Brazález B, Romay-Barrero H. Manual Lymph Drainage With Progressive Arm Exercises for Axillary Web Syndrome After Breast Cancer Surgery: A Randomized Controlled Trial. Phys Ther. 2022 Mar 1;102(3):pzab314. doi:* 10.1093/ptj/pzab314. *PMID: 35079831.*
- Page 2, line 6 starting with “apy [7,8]. Pneumatic multicompartmental…” should read “apy [6,7]. Pneumatic multicompartmental…”
- Page 9, Table 3 Study variables, should read “x” on “Arm Volume” for the article Torres et al. 2022.
- Page 9, Table 4 PEDro Scale, should show as follows:

**Table:** 

Study	Total Score	1	2	3	4	5	6	7	8	9	10	11
Torres et al, 2022 [23]	7/10	-	x	-	x	-	-	x	x	x	x	x
Muñoz et al, 2021 [24]	4/10	-	-	-	x	-	-	-	-	x	x	x
Klein et al, 2021 [25]	7/10	-	x	x	x	-	-	-	x	x	x	x
Nirmiti et al, 2019 [30]	5/10	-	x	-	-	-	-	-	x	x	x	x
Gamal Abd et al, 2018 [31]	6/10	-	x	-	x	-	-	-	x	x	x	x
Ibrahim et al, 2018 [26]	5/10	-	x	-	x	-	-	-	-	x	x	x
Ochalek et al, 2017 [27]	7/10	-	x	-	x	-	-	x	x	x	x	x
Cho et al, 2016 [28]	5/10	-	x	-	-	-	-	x		x	x	x
Yuste, 2015 [29]	6/ 10	-	x	x	-	-	-	x		x	x	x

- Page 12, paragraph 3 starting with “There are two systematic reviews similar…”, should read instead “ There is a narrative review and a systematic review similar to this one [36,10]”.
- Page 12, paragraph 4 starting with “Some studies [27-30] were discarded…”, should read instead “Some studies [31, 33, 27, 26] were discarded…”.

